# Pericardial Epithelioid Inflammatory Myofibroblastic Sarcoma: An Atypical Presentation

**DOI:** 10.7759/cureus.26827

**Published:** 2022-07-13

**Authors:** Mazieyar Azad, Melissa Oye, Natalie Torrente, Mehdi Mirsaeidi

**Affiliations:** 1 Neurology, University of Florida, College of Medicine, Jacksonville, USA; 2 Internal Medicine, University of Florida, College of Medicine, Jacksonville, USA; 3 Pulmonary and Critical Care Medicine, University of Florida, College of Medicine, Jacksonville, USA

**Keywords:** sarcoma, immunohistochemical stain, pericardium, cancer, epithelioid inflammatory myofibroblastic sarcoma

## Abstract

Sarcomas are a diverse group of cancers of mesenchymal origin. Epithelioid inflammatory myofibroblastic sarcoma (EIMS) is an uncommon and hardly reported neoplasm that is a malignant variant of the typically benign inflammatory myofibroblastic tumor (IMS). We discuss an exceedingly rare case of a 53-year-old patient with primary EIMS of the pericardium who presented in impending hemodynamic collapse. A transthoracic echocardiogram revealed a large circumferential pericardial effusion with tamponade physiology and an echogenic intrapericardial mass compressing the right ventricle to near obliteration. She underwent emergent sternotomy with resection and one cycle of chemotherapy with liposomal doxorubicin before having recurrent metastatic pericardial and pleural effusions, ultimately leading to her unfortunate passing.

## Introduction

Sarcomas are a diverse group of cancers of mesenchymal origin that comprise approximately 11% of all cancers [1]. Cancer is the second leading cause of death in the United States, with a reported 602,350 cancer deaths in 2020 [2]. Sarcomas can present a diagnostic challenge to pathologists due to their heterogeneity. Epithelioid inflammatory myofibroblastic sarcoma (EIMS) is a rare and aggressive form of sarcoma. EIMS is a variant of inflammatory myofibroblastic tumor (IMT) that has malignant behavior and is associated with a poor prognosis. The World Health Organization defines IMT as an intermediate soft tissue tumor composed of myofibroblast-differentiated spindle cells accompanied by numerous inflammatory cells, plasma cells, and/or lymphocytes [3]. 

EIMS is distinguished by its morphological and histological elements, including a predominance of epithelioid cells with vesicular nuclei in a setting of myxoid stroma, inflammatory cell infiltrates, and the presence of spindle cells [4]. In addition, it typically has a perinuclear or nuclear membrane staining pattern with anaplastic lymphoma receptor tyrosine kinase gene (ALK) [4,5]. These tumors are often intra-abdominal and more aggressive than conventional inflammatory myofibroblastic tumors [4,6]. These clinicopathological features have been noted in many cases of EIMS and have served as indicators of malignant potential and poor prognosis. In many cases, local recurrences and fatalities are reported despite surgical resection, chemotherapy, and radiation therapy [6]. Marino-Enriquez et al. proposed the designation of EIMS to define a distinctively intra-abdominal, highly malignant variant of inflammatory myofibroblastic sarcoma [6]. However, there have been several cases in the literature of extra-abdominal sites of EIMS [7,8]. Variations of EIMS with atypical clinicopathological features may present a diagnostic challenge for both clinicians and pathologists. Such variations may have implications for the efficacy of proposed novel targeted therapies as well. We present a case of confirmed EIMS with a negative staining pattern for ALK manifesting in the pericardium.

## Case presentation

**Figure 1:**
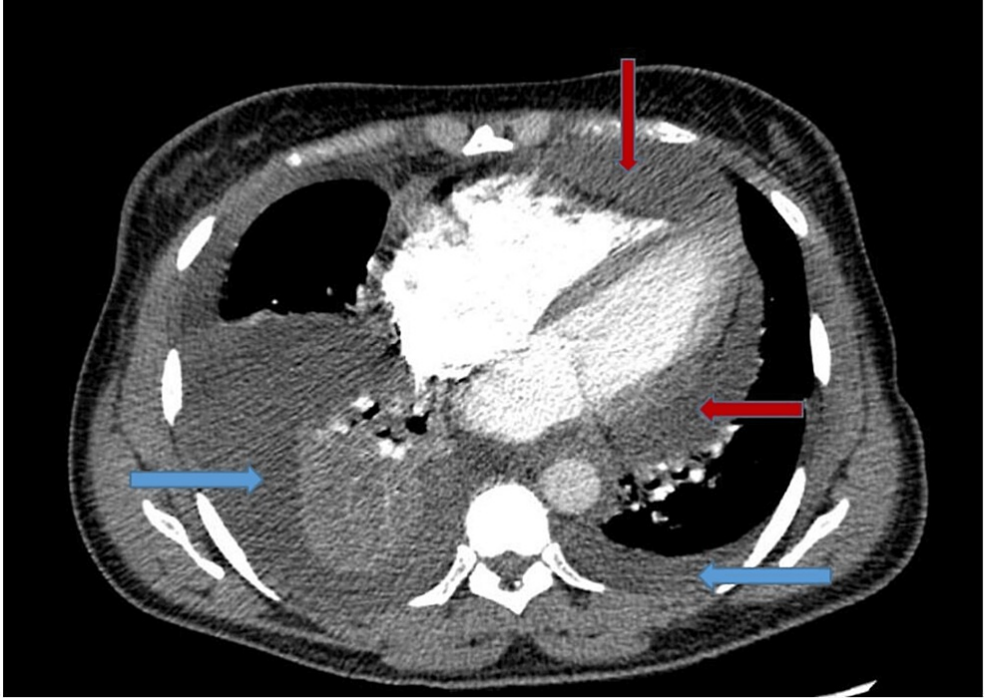
CT of chest showing a large pericardial effusion (red arrows), a large right-sided pleural effusion, and a small left-sided pleural effusion (blue arrows)

**Figure 2:**
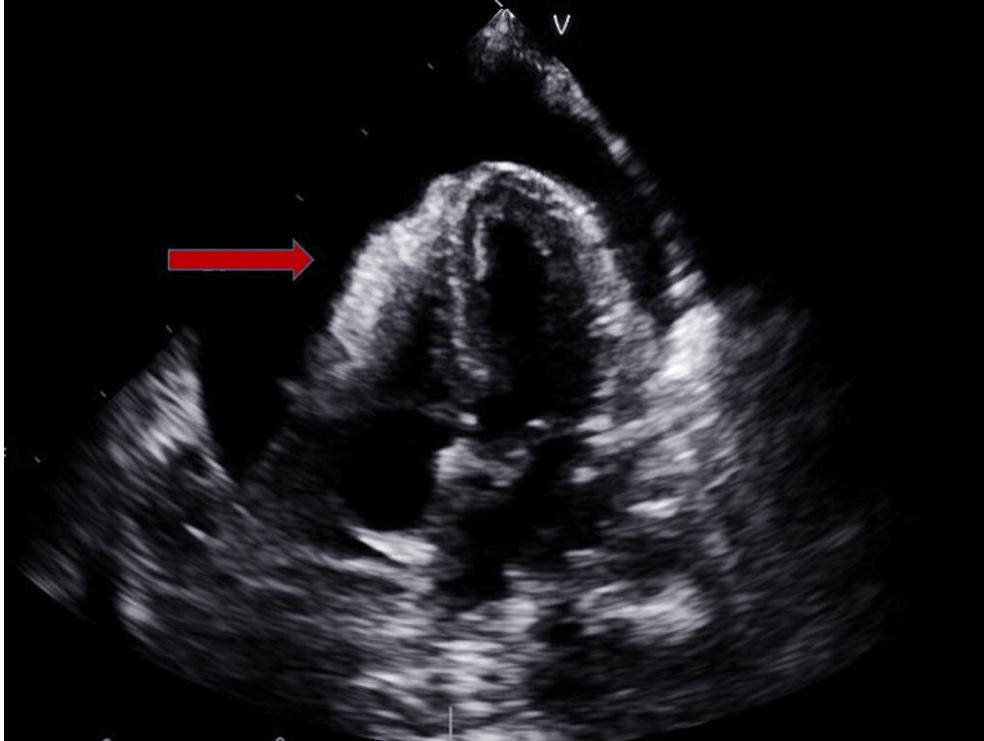
TTE showing a large pericardial effusion (red arrow) TTE - transthoracic echocardiogram

**Figure 3:**
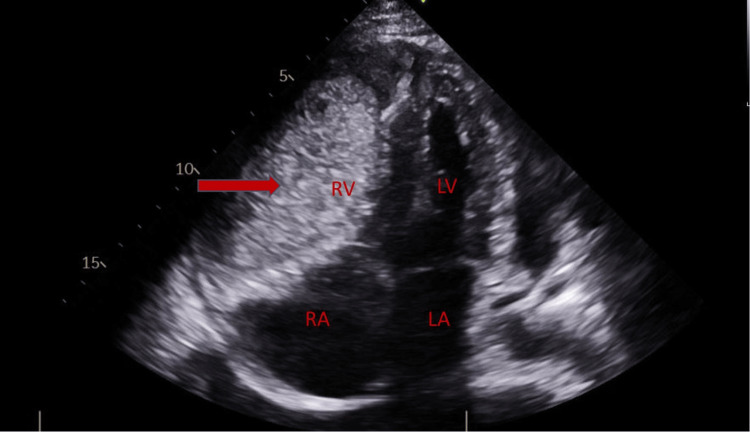
TTE showing an echogenic intrapericardial mass compressing the right ventricle to near obliteration (red arrow) The chambers of the heart are labeled. TTE - transthoracic echocardiogram; RV - right ventricle; LV - left ventricle; RA - right atrium; LA - left atrium

**Figure 4:**
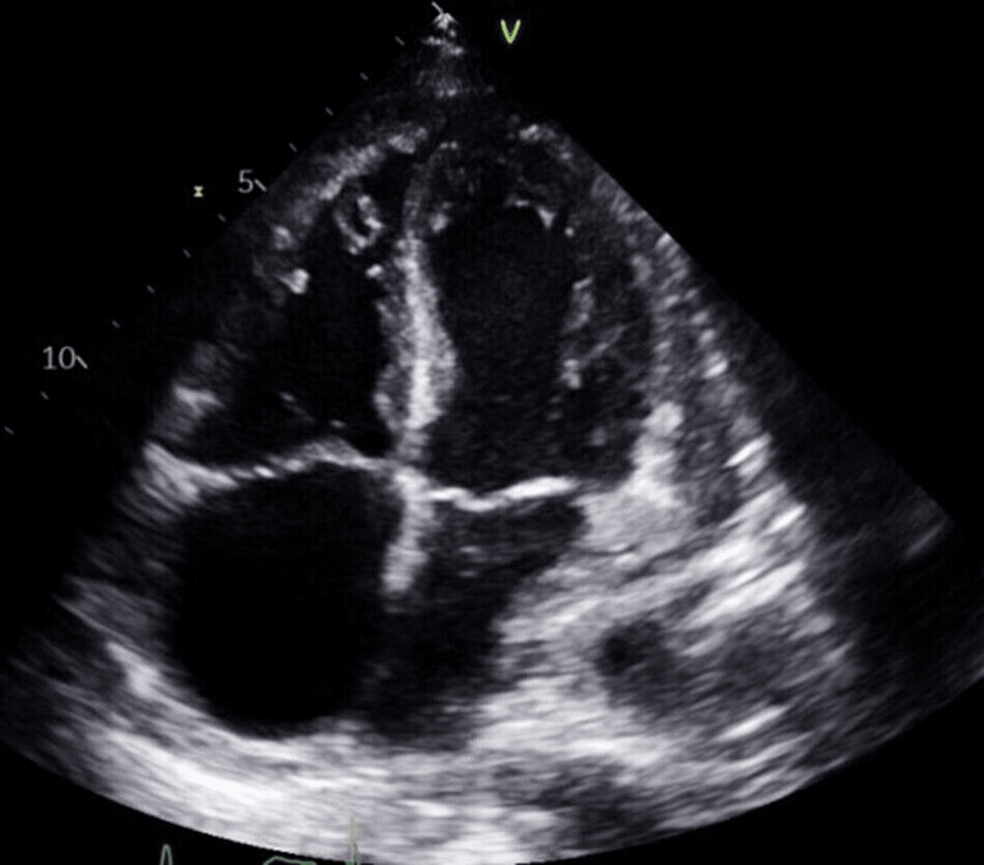
Postoperative TTE showing resolution of the prior pericardial effusion TTE - transthoracic echocardiogram

A 53-year-old woman with a past medical history of factor V Leiden mutation and multiple prior venous thromboembolisms with inferior vena cava filter placement presented with progressive fatigue and shortness of breath. On exam, she was in visible respiratory distress, hypoxic, and tachypneic. A computed tomography (CT) angiogram of the chest revealed bilateral pleural effusions and a large circumferential pericardial effusion (Figure 1). A transthoracic echocardiogram (TTE) revealed a large circumferential pericardial effusion with tamponade physiology and an echogenic intrapericardial mass compressing the right ventricle to near obliteration (Figures 2-3). She was taken to the operating room for an emergent sternotomy with extensive decortication and evacuation of an intrapericardial hematoma. Postoperative TTE showed resolution of the pericardial effusion (Figure 4). Biopsy showed a malignant tumor composed primarily of epithelioid cells with vesicular nuclei, large nucleoli, and amphophilic cytoplasm in a background of abundant myxoid stroma containing mixed neutrophils and lymphocytes as well as spindle components. Immunohistochemical staining (IHC) was positive for AE1/AE3, desmin, CD30, and D2-40 and negative for ALK1. Next-generation sequencing panel performed at an outside institution detected no gene fusions. The final diagnosis was an EIMS of the pericardium. After recovery from surgery and discharge home with significant symptomatic improvement, she was started on chemotherapy with liposomal doxorubicin.

**Figure 5:**
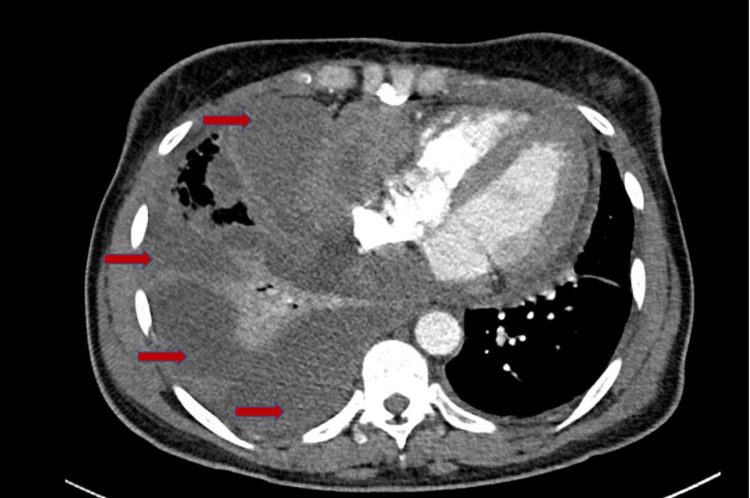
CT of chest showing multiple, large multi-loculated pleural effusions of the right hemithorax causing complete opacification (red arrows)

Only two months later, she was admitted to the medical intensive care unit for acute hypoxic respiratory failure requiring endotracheal intubation. CT of chest angiogram revealed multiple, large multi-loculated pleural effusions of the right hemithorax, causing complete opacification (Figure 5). She underwent CT-guided thoracentesis with the placement of multiple pleural drains. Cytology from the pleural fluid was consistent with metastatic EIMS. A repeat TTE showed findings suggestive of cardiac tamponade from a pericardial tumor compressing on the right ventricle with minimal to moderate pericardial fluid. She was deemed a poor candidate for any surgical intervention. She had received one cycle of chemotherapy, and given her poor clinical status, she could not be offered continued systemic chemotherapy. Per patient and family wishes, all aggressive interventions were withdrawn, and comfort care was pursued.

## Discussion

EIMS is a rare and recently defined entity that represents a more aggressive subtype of inflammatory myofibroblastic tumor (IMT). There have been several reports in recent years describing numerous cases of intra-abdominal EIMS, with a smaller number of cases being extra-abdominal, primarily in the thoracic or mediastinal spaces [4]. The majority of these cases report positive staining for ALK receptor tyrosine kinase, and many cases also report associated RRBP1-ALK and RANBP2-ALK gene fusions, which distinguish EIMS from traditional inflammatory myofibroblastic sarcomas [4,5]. We reported a case of pericardial EIMS showing atypical genetic markers. Histological examination of the tumor was the key for diagnosis in this patient.

The diagnosis of EIMS can be difficult owing to its rarity and resemblance to other malignant diseases of mesenchymal or lymphoproliferative origins [9]. In our case, the diagnosis was established once histological evaluation revealed the typical features that characterize the epithelioid subtype of inflammatory myofibroblastic sarcoma, namely a predominance of epithelioid cells with atypical nuclei in the presence of myxoid stroma containing inflammatory cells and spindle cells.

Cardiac EIMS, specifically of pericardial origin, is exceedingly rare. The largest review of patients with cardiac IMT was of 57 patients performed by Eilers et al. [10]. In their study, cardiac IMT had a pediatric predilection, with the average age being 19 years old. The most common presentation was cardiac, including chest pain, heart failure, myocardial infarction, and/or arrhythmias [9]. There are a few reported cases of sudden cardiac death found to be secondary to cardiac IMT on autopsy [11,12]. Although the incidence of EIMS is low, it remains an aggressive tumor with a poor prognosis.

Clinician and pathologist familiarity with this disease can be crucial for diagnosis. It is important for clinicians to be aware of variations in the presentation of EIMS and for pathologists to be aware of the characteristic histological features as the course of the disease is often aggressive and rapidly fatal. In addition, proposed therapies that target specific markers typical of EIMS should consider variants that do not express the most common markers. Du et al. present one example of a proposed therapy that could target cases that express PD-L1 [9]; however, such expression was not reported in our case. Variants of EIMS that do not express the common genetic markers may provide a therapeutic challenge for such proposed targeted therapies.

## Conclusions

EIMS is a rare and aggressive cancer that has conventionally been recognized by its intra-abdominal presence and distinctive histological and genetic characteristics. Our case demonstrates that pericardial manifestations and ALK-negative presentations are possible with EIMS. This sarcoma can be a challenge for both clinicians and pathologists to diagnose. Knowledge of more atypical presentations can aid in earlier diagnoses. In the future, targeted therapies should consider variants of EIMS that do not express the typical genetic markers as this may affect prognosis.
